# Evaluation of the significance of intermediate coronary artery stenoses by stress perfusion CMR

**DOI:** 10.1186/1532-429X-13-S1-P107

**Published:** 2011-02-02

**Authors:** James D Richardson, Angela G Bertaso, Hussam Tayeb, Dennis Wong, Adam J Nelson, Benjamin K Dundon, Payman Molaee, Kerry Williams, Matthew I Worthley, Karen S Teo, Stephen G Worthley

**Affiliations:** 1 Cardiovascular Research Centre, Royal Adelaide Hospital & Department of Medicine, University of Adelaide, Adelaide, Australia; 2 Cardiovascular Research Centre, Royal Adelaide Hospital, Adelaide, Australia

## Objective

To determine the feasibility of adenosine stress cardiac magnetic resonance (CMR) to guide clinical management of patients with a documented intermediate angiographic coronary stenosis.

## Background

A number of patients undergoing assessment for possible ischaemic heart disease proceed straight to coronary angiography without prior non-invasive functional tests. When an intermediate coronary lesion is then encountered, the functional significance of that lesion is often unclear. Invasive assessment with fractional flow reserve can clarify the situation but is not always available and involves significant expense. Non-invasive tests are then frequently requested to guide treatment. Adenosine stress perfusion imaging has been shown to have a high sensitivity and specificity for detecting ischaemic heart disease. We sought to determine the ability of stress perfusion CMR to guide management in these patients.

## Methods

Retrospective analysis of patients with an intermediate coronary stenosis on angiogram subsequently referred for adenosine stress CMR. Patients with previous CABG were excluded. Only those with single vessel disease were evaluated. All intermediate lesions were in the range 30-70% on angiogram. Perfusion imaging was obtained at stress (adenosine 140 µg/kg/min) and rest on a 1.5T scanner. Late enhancement was assessed with dual pass gadolinium (0.2mmol/kg total dose). Patient records, hospital databases and national death registries were reviewed. We defined MACE as cardiovascular death, myocardial infarction, revascularisation, and ischaemic hospitalisation.

## Results

Thirty nine patients, with follow up data for a median 25 months (IQR 22-28), were identified. Of those with a positive adenosine stress CMR, 69% (9 of 13) encountered MACE. These were all PCI events. Of those with a negative CMR, MACE occurred in 12% (3 of 26). These were also PCI events, however only two of these were symptom driven, due to lesion progression at 9 and 10 months respectively.

## Conclusions

Adenosine stress perfusion CMR may be a useful tool in the clinical management of patients with an intermediate coronary artery stenosis.

